# Prospective randomized multicenter noninferiority clinical trial evaluating the use of TFN-advanced^TM^ proximal femoral nailing system (TFNA) for the treatment of proximal femur fracture in a Chinese population

**DOI:** 10.1007/s00068-023-02231-x

**Published:** 2023-02-13

**Authors:** Lidan Zhang, Zhijun Pan, Xiaohui Zheng, Qiugen Wang, Peifu Tang, Fang Zhou, Fan Liu, Bin Yu, Frankie K. L. Leung, Alex Wu, Suzanne Hughson, Zhuo Chen, Michael Blauth, Anthony Rosner, Charisse Sparks, Manyi Wang

**Affiliations:** 1 Beijing Jishuitan Hospital, No.31 Xinjiekou East Road, Xicheng District, Beijing City, China; 2 The Second Affiliated Hospital of Zhejiang University School of Medicine, No. 88 Jiefang Road, Hangzhou City, Zhejiang Province China; 3 The First Affiliated Hospital of Guangzhou University of Chinese Medicine, No.16 Baiyun Airport Road, Guangzhou City, Guangdong Province China; 4 Shanghai General Hospital, No.100 Haining Road, Hongkou District, Shanghai City, China; 5 Chinese PLA General Hospital, No. 28 Fuxing Road (Wukesong), Haidian District, Beijing City, China; 6 Peking University Third Hospital, Garden North Road No.49, Haidian District, Beijing City, China; 7 Affiliated Hospital of Nantong University, No. 20 Xisi Road, Nantong City, Jiangsu Province China; 8 Nanfang Hospital of Southern Medical University, No. 1838 North of Guangzhou Ave, Baiyun District, Guangzhou City, Guangdong Province China; 9 The University of Hong Kong Shenzhen Hospital, No. 1 Haiyuan Road, Futian District, Shenzhen City, China; 10 DePuy Synthes Products, Inc., 1310 Goshen Parkway, West Chester, PA 19380 USA; 11 Medical Affairs, Johnson & Johnson Medical (Shanghai) Ltd., 15F, Tower 3 China Central Place, No.77, Jianguo Road, Chaoyang District, Beijing City, China; 12 Medical Affairs, Synthes GmbH, Solothurn, Luzernstrasse 21, 4258 Zuchwil, Switzerland

**Keywords:** Trochanteric hip fracture, Cephalomedullary nail, Trochanteric Fixation Nail Advanced (TFNA), Proximal Femoral Nailing Antirotation II (PFNA-II), Fracture union rate, Randomized controlled trial, Noninferiority trial

## Abstract

**Purpose:**

To evaluate whether the 24-weeks postoperative fracture union rate for the investigational TFNA intramedullary nail was non-inferior compared to the control product PFNA-II.

**Methods:**

The study was a prospective, randomized, single-blind, noninferiority dual-arm study drawing from 9 trauma centers across China, between November 2018 and September 2020, with follow-up measurements at 24 weeks after internal fixation. The full analysis data set (FAS [Intent-to-Treat]) was analyzed and is summarized here. The primary outcome was fracture union rate, a composite score combining clinical and radiographic assessment. Secondary endpoints comprised (a) clinical outcomes including (1) SF-12, (2) Harris Hip, and (3) EQ-5D Scores, (b) radiographic incidence of complications such as loosening or cut-out requiring revision, (c) revision rates, (d) reoperation rates, and (e) adverse events, including 24-weeks revision and reoperation rates.

**Results:**

Both TFNA and PFNA-II group fracture healing rates were 100% at 24 weeks; TFNA was therefore shown to be non-inferior to PFNA-II. With baseline data matched in all parameters except age in both the TFNA and PFNA-II groups, comparisons of union rates, SF-12, Harris Hip, and EQ-5D Scores yielded *p* values > 0.05 indicating no significant difference between the two groups, further supporting the noninferiority of TFNA. In both groups, revision and re-operation rates were 0, and the incidences of serious adverse events were 19.4% and 17.4%, respectively.

**Conclusion:**

In terms of fracture union rate at 24 weeks, the DePuy Synthes Trochanteric Fixation Nail Advanced (TFNA) was not inferior to the marketed Proximal Femoral Nail Antirotation (PFNA-II) device produced by the same manufacturer. Secondary and safety outcomes showed no significant differences between the two groups.

**Registration:**

Registration was completed at ClinicalTrials.gov NCT03635320.

## Introduction

Several types of cephalomedullary nails are commercially available. The Proximal Femoral Nailing Antirotation (PFNA; DePuy Synthes, Oberdorf, Switzerland) manufactured from a titanium alloy (Ti-6Al-7Nb) is an advanced version of the previous PFN nail featuring an anti-rotation helical blade and currently available for clinical use in China. The PFNA-II, also available in China, is a smaller version of the PFNA designed for the reduced femur size of typical Asian populations [1].

The TFN-Advanced^TM^ Proximal Femoral Nailing System (TFNA, DePuy Synthes, Oberdorf, Switzerland) was introduced in 2015 with a number of updated design features: (a) a reduced proximal nail diameter, (b) a reduced radius of curvature, (c) an oblique cut of the lateral end of the helical blade or screw, (d) provision of a higher strength titanium alloy T-15Mo (TiMo), (e) the introduction of static, dynamic or oblique locking options, and (f) a variety of features to increase procedural efficiency. These design features were intended to address unmet needs, offering the possibilities of (a) reducing cut-out by facilitating the compaction of cancellous bone surrounding the implant [2], (b) reducing anterior impingement, (c) preventing lateral protrusion, (d) reducing nail breakage, (e) preventing distal impingement (due to curvature misalignment), and (f) facilitating surgical procedures with the possibility of abbreviating operating time.

The PFNA-II was selected as the comparator device for this study. The PFNA-II is an appropriate reference product for the TFNA, as its intended use as well as clinical, technical, and biological parameters are similar. The intended uses of PFNA-II and TFNA are the treatment of proximal femur fractures and combination proximal and shaft fractures of the femur.

This study was undertaken to collect pre-market data on TFNA to support a marketing application in China. The goal was to compare the effectiveness and safety of the TFNA system with the commercially available PFNA-II for the treatment of proximal femoral trochanteric fractures. The primary objective was to evaluate fracture union at 24 weeks postoperatively with secondary measures pertaining to the quality of life and safety. In terms of convenience and presumed time and cost savings, the noninferiority trial was designed according to specific, established protocols [3, 4] in recognition that its use has increased in recent years [5, 6]. The assumption was that matching outcomes of the TFNA and PFNA-II products, using the latter as a standard of care, represented a desirable demonstration of noninferiority.

## Methods

### Study design

One hundred eighty-eight participants were enrolled, beginning November 2018 and concluding September 2019 across nine sites in China. The last patient visit occurred May 2020, and the database was locked September 2020. The ethics committees approved the protocol and informed consent at each participating site. The study was registered on ClinicalTrials.gov (identifier: NCT03635320). Eligible men and women were aged 18 years or older and had sustained a unilateral proximal femur fracture (AO 31-A1, 31-A2, 31-A3), or trochanteric fracture with diaphyseal extension. Key exclusion criteria included fractures where the operative treatment occurred 3 weeks after surgery, femoral head and neck fractures (AO 31-B and 31-C), pathologic fracture, revision surgeries, serious soft tissue injury, and multiple systemic injuries. The complete list of inclusion and exclusion criteria is found in Table 1. All patients were followed for 6 months after time of surgery.

**Table 1:** Inclusion and exclusion criteria

Inclusion	Exclusion
Age ≥ 18 years	Patients did not provide voluntary consent to participate in study
Unilateral proximal femur fracture treated with intramedullary nail internal fixation	Patients were pregnant or lactating
Fracture type classified as pertrochanteric (31-A1 and 31-A2), intertrochanteric (31-A3) or trochanteric with diaphyseal extension (31-A1/A2/A3)	Operations were performed > 3 weeks after the primary injury
Ability to speak and understand questions in patient reported outcomes with response in an understandable language	Patients presented with femoral head and/or neck fractures (31-B and 31-C)
	Pathological features such as primary or metastatic tumor
	Serious soft tissue injury was deemed by investigator to impact fracture union; presence of combined vascular injury, or combined osteofascial compartment syndrome
	Multiple systemic injuries judged by investigators as not suitable for enrollment, or orthopedic fractures in other bones at three or more sites
	Revision surgeries (e.g., malunion, nonunion, or infection)
	Concurrent medical conditions such as diabetes, metabolic bone disease, post-polio syndrome, poor bone quality, or prior history of poor fracture union
	Patients with anesthetic and/or surgical contraindications
	Patients known to be allergic to implant components
	Patients who were using chemotherapeutics or accepting radiotherapy, using systemic corticosteroid hormone or growth factor, long-term use of sedative hypnotics (continuous use over 3 months) or non- steroidal anti-inflammatory medications (continuous use over 3 months
	Patients exhibited potential bad compliance issues such as dementia, schizophrenia, excessive drinking, smoking, or drug abuse
	Patients who participated in other clinical trials over the previous 3 months prior to consent

Patients enrolled at each study site were informed in advance that they would be randomized in a 1:1 ratio to either the investigational group (TFNA) or the control group (PFNA-II) using a separate block group randomized schedule at each study site, ensuring the consistent distribution of patients in the treatment group and control group. Eligible patients were randomly assigned to the TFNA, or PFNA-II study group using a centralized computer-generated randomization system (Medidata Balance, Dassault Systèmes, Shanghai). Patients were blinded to the treatment they had received until study completion; study personnel were not blinded. Patients and investigators were unaware of block sizes.

The study’s null and alternative hypotheses were as follows:

$$\begin{gathered} {\text{H}}_{{\text{o}}} :{\text{ P}}_{{\text{PFNA - II}}} - {\text{ P}}_{{{\text{TFNA}}}} \geq {1}0\% , \hfill \\ {\text{H}}_{{\text{a}}} :{\text{P}}_{{\text{PFNA - II}}} {-}{\text{P}}_{{{\text{TFNA}}}} < {1}0\% , \hfill \\ \end{gathered}$$

where P_PFNA-II_ represents the fracture union rate among those receiving the PFNA-II implant, P_TFNA_ represents the fracture union rate among those receiving the TFNA implant, and 10% is the pre-specified non-inferiority margin [7].

### Radiographic assessments

Radiographic film review was conducted by two independent reviewers with a third reviewer involved to resolve discrepancies and render a final decision. At the preoperative visit, the investigator documented the fracture type according to the AO classification system [8]. Based on postoperative x-rays, the independent radiologist determined whether there was radiographic evidence of a secondary surgical procedure (for example, head element removal). The radiologist also provided assessment of the implant status and whether the device was in good condition or if any mechanical complications had occurred, including the qualitative assessment of the fracture line and callus.

### Sample size

In order to achieve 80% power for a non-inferiority evaluation, the assumption was that there was no difference between the fracture union rate of the two groups; anticipated fracture union rates were assumed to be 95%, and the non-inferiority margin was 10% by using the one-sided significance level of 0.025 [9]. One hundred fifty patients (75 in each group) were required for analysis. Considering a 20% drop-out caused by unexpected circumstances (patient deaths, withdrawals from the study, loss to follow-up) and further strengthening the safety evaluation of the study, 188 patients (94 patients in each group) were targeted for enrollment.

### Surgical procedure

Surgeons recruited in the study had previous experience with cephalomedullary nailing. The surgical procedure was a closed reduction (and/or open reduction if needed) under fluoroscopy. The implantation of both nail systems is similar involving opening of the medullary cavity, insertion of the nails and head assembly, distal locking, and insertion of the end cap (if desired). While the TFNA is available with both screw and blade head elements, the PFNA II is only available with a helical blade head element.

### Primary endpoint

The primary endpoint was fracture union at 24 weeks postoperatively. Successful fracture union was a composite end point. For individual patients, the treatment was considered successful if all of the following conditions were met 24 weeks after surgery: (a) no focal tenderness or lengthwise percussion pain, or abnormal movement; (b) the frontal/lateral X-ray examination showed vague or no fracture gap, or continuous callus passing across the fracture line; and (c) no deformation or breakage was found in the implants. Representative case examples of AO/OTA 31A1-3 fractures with uneventful healing with PFNA-II or TFNA are shown in Fig. 2.

### Secondary endpoints

Secondary endpoints included (a) clinical outcomes: SF-12, Harris Hip Score, and EQ-5D, (b) adverse events (type and frequency) compared between investigational and control groups, (c) 24-weeks revision rate (removal of any component for any reason), and (d) 24-weeks reoperation rate (secondary surgery at the fracture site[s] performed for any reason). In addition, assessments were collected on the surgeon’s evaluation of product operability during the index procedure.

SF-12 scores were summarized for both treatment groups at baseline, and at the 12- and 24-weeks follow-up visits. In addition, SF-12 scores were summarized for change from baseline. An ANCOVA model was used with the change from baseline as the outcome variable. The treatment group was an independent variable, and the baseline value was the concomitant variable. The Harris Hip Score was only collected at the 12- and 24-weeks follow-up visits. The 12- and 24-weeks scores were summarized and compared between groups using the t test. Qualitative outcomes were compared between groups using either a Chi-square test or Fisher’s exact probability test. The EQ-5D Health Index (HI) was based upon Japanese population coefficients.

### Safety

In this study, adverse events (AEs) were defined as any untoward medical occurrence, unintended disease or injury, or untoward clinical signs (including abnormal laboratory findings) in patient, users, or other persons, whether or not related to the investigational medical device. Serious adverse events (SAEs) were defined as any AE that (a) led to death; (b) led to serious deterioration in the health of the patient that either resulted in (1) a life-threatening illness or injury, or (2) a permanent impairment of a body structure or a body function, or (3) in-patient or prolonged hospitalization, or (4) medical or surgical intervention to prevent life-threatening illness or injury or permanent impairment to a body structure or body function; (c) led to fetal distress, fetal death or a congenital abnormality or birth defect. At each evaluation of the patient enrolled in the clinical investigation, the investigator determined whether any AE or SAE occurred and determined its possible relationship to the study devices or procedure. Adverse events were coded and summarized by MedDRA system organ class (SOC) and preferred term (PT). Causal relationships of AEs to the device were rated as unrelated, unlikely, possible, probable, or related. Adverse events were collected regardless of the relationship to the procedure or the device.

### Baseline variables

Baseline variables were ascertained after randomization with self-reported questionnaires and with a physical examination performed by a trained research assistant.

### Statistical analysis

**Fig. 1:**
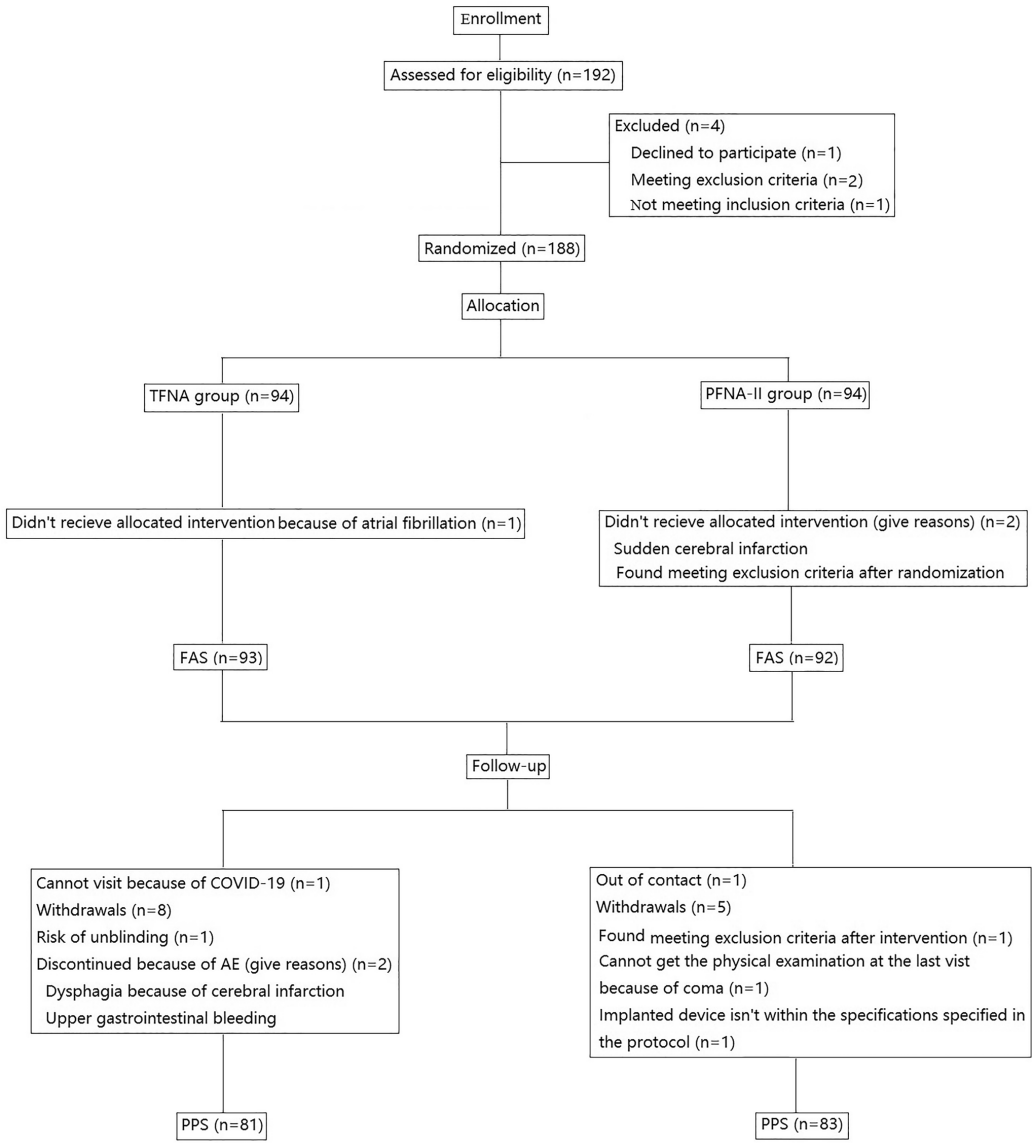
Flow diagram showing patient enrollment and follow-up. Abbreviations: *FAS* Full Analysis Set, *PPS* Per Protocol Set, *PFNA-II* Proximal Femoral Nailing Antirotation, *TFNA* Trochanteric Fixation Nail Advanced, *n* number

The analysis populations consisted of a full analysis set (FAS) in which the data set constituted all patients who were consented and received an implant, and a Per Protocol Set (PPS), which comprised a set of patients that completed the trial but excluded those with a serious protocol violation affecting the primary efficacy endpoint (Fig. 1). The results from the FAS are presented, as utilization of the FAS is appropriate for all of the outcome comparisons evaluated here.

Patient demographics and baseline variables were provided for the FAS population. Descriptive statistics for continuous variables included the number of patients, mean, and standard deviation. Descriptive statistics for dichotomous/categorical variables included number and percent of patients. A two-sided alpha of 0.05 was used for statistical testing and confidence intervals unless otherwise noted.

For calculating the confidence interval when both treatment and control groups were binomial extremes (0% and 100%, fractured and resolved), the Newcombe–Wilson method was applied [10].

## Results

### Eligibility

One hundred ninety-two patients were screened, and 188 patients were included and randomly assigned to a group; enrolment included 94 in the investigational group and 94 in the control group. Three patients were not implanted with a device, resulting in 185 patients in the FAS. Accounting for drop-outs, there were 164 patients in the PPS, including 81 in the TFNA group and 83 in the PFNA-II group. Reasons for exclusion prior to final assignments to the FAS and PPS groups are shown in Fig. 1. With all outcomes closely matched between the PPS and FAS populations, the FAS data are presented for the remainder of the report.

### Baseline condition

There were no significant baseline differences in demographic data or fracture characteristics except age (*p*=0.0274) of patients between the study group and the control group. There were no crossovers (patients assigned to one group who received a device from the other). For the TFNA group, the average age was 77.8 ± 10.80 including 29 males and 64 females, while for the PFNA-II group, the average age was 74.1 ± 11.80 including 33 males and 59 females. Statistical differences regarding gender, and BMI were not significant (Table 2).

**Table 2:** Patient and fracture characteristic (FAS)

Index	Study group *N* = 93	Control group *N* = 92	*p* value
*Demographics*			
Age (year)			
Mean ± SD	77.8 ± 10.80	74.1 ± 11.80	0.0274
Gender			
Male	29 (31.2%)	33 (35.9%)	0.4994^a^
Female	64 (68.8%)	59 (64.1%)	
Weight (kg) Mean ± SD	59.34 (13.84)	60.22 (12.53)	0.6494^b^
Height (cm) Mean ± SD	160.4 (8.46)	161.6 (8.15)	0.3277^b^
BMI (kg/m^2^)	22.92 (4.28)	22.93 (3.73)	0.9923^b^
*Fracture characteristics*			
Fracture site: lower limbs			0.9378^a^
Left	49 (52.7%)	49 (53.3%)	
Right	44 (47.3%)	43 (46.7%)	
Type of trochanteric fracture			0.4312^a^
31 A1	43 (46.2%)	41 (44.6%)	
31 A2	42 (45.2%)	47 (51.1%)	
31 A3	8 (8.6%)	4 (4.3%)	
Type of fracture			
Open	0	0	NA
Closed	93 (100%)	92 (100%)	
Closed Fracture Tscherne Type			0.6663^c^
C 0	85 (91.4%)	87 (94.6%	
C I	7 (7.5%)	5 (5.4%)	
C II	1 (1.1%)	0	
C III	0	0	
Reason for fracture			0.3603^c^
High energy	2 (2.2%)	5 (5.4%)	
Low energy	90 (96.8%)	87 (94.6%)	
Presence of other fractures^d^			0.5626^a^
Yes	7 (7.5%)	5 (5.4%)	
No	86 (92.5%)	87 (94.6%)	

Abbreviations: *BMI* Body Mass Index, *cm* centimeter, *kg* kilogram, *m* meter, *N* number, *SD* standard deviation

^a^Likelihood ratio Chi-square test

^b^*t*-test

^c^Fisher’s exact probability test

^d^Fractures in < 3 other bones; study had exclusion criteria for fractures in other bones at ≥ 3 sites

### Surgery and fracture details

All fractures were closed, and the reason for fracture was low energy in 96.8% of cases for TFNA and 94.6% of cases for PFNA-II. Most fractures in both groups were either 31-A1 or 31-A2 (Table 2). Closed reduction was used for all fractures in the TFNA group and for all but one fracture in the PFNA-II group. Reaming was used in 5 (5.4%) cases in the TFNA group and in 9 (9.8%) cases in the PFNA-II group (Table 3); this difference was not significant (*p*=0.254). The difference in intraoperative blood loss between the study (131.0 mL ± 97.4 mL) and control (130.9 mL ± 126.3 mL) groups was insignificant (*p*=0.9953), as was surgery time (1.22 h ± 0.49 hr and 1.08 h ± 0.56 h) for the respective groups *(p*=0.0610) (Table 3).

**Table 3:** Surgery characteristics and evaluation of product operability and tools

Index	Study group *N*= 93	Control group *N*=92	*p* values
*Operating characteristics*			
Blood loss during surgery (mL)	131.0 ± 97.4	130.9 ± 126.3	0.9953^a^
Surgery time (hr)	1.22 ± 0.49	1.08 ± 0.56	0.0610^a^
Reaming performed (*n*/%)	5 (5.4%)	9 (9.8%)	0.254^b^
Method of fracture reduction			
Closed	93 (100%)	92 (98.9%)	
Open	0	1 (1.1%)	
*Operability*			
Difficulty in determining nail position			0.1210^c^
Good	93 (100%)	89 (96.7%	
Average	0	3 (3.3%)	
Difficulty in inserting an IM nail			0.2459^c^
Good	93 (100%)	90 (97.8%	
Average	0	2 (2.2%)	
Difficulty level of proximal locking			0.6209^c^
Good	92 (98.9%)	90 (97.8%)	
Average	1 (1.1%)	3 (2.2%)	
Difficulty level of distal locking			1.000^c^
Good	90 (96.8%)	89 (96.7%	
Average	3 (3.2%)	3 (3.3%)	
Difficulty level of placing end caps^d^			0.1315^c^
Good	82 (91.1%)	71 (82.6%	
Average	8 (8.9%)	13 (15.1%)	
Poor	0	2 (2.3%)	
Impact of IMN placement on restoration			0.6823^c^
No impact	91 (97.8%)	89 (96.7%)	
Better than before	2 (2.2%)	3 (3.3%)	
Tools in the toolbox			0.4969^c^
Good	87 (93.5%)	89 (96.7%)	
Average	6 (6.5%)	3 (3.3%)	

Abbreviations: *hr* hour, *IMN* intramedullary nail, *mL* milliliter, *N* number

^a^*t*-test

^b^Likelihood ratio Chi-square test

^c^Fisher’s exact probability test

^d^*N* = 90 Study Group; *N* = 86 Control Group

Regarding the ease of operability of the two nail systems, there were no significant differences in the difficulties of determining nail position, nail insertion, proximal or distal locking, or placement of end caps. In addition, the impact of nail placement on maintenance of the reduction and surgeon satisfaction with the instruments available in the sets did not show a significant difference between groups (Table 3).

As discussed above, the TFNA is available with both screw and blade head elements, and the PFNA II is only available with a helical blade head element. With regard to the proximal head element, 80 patients received blades and 13 patients received screw head elements in the TFNA group. All patients in the PFNA-II group received helical blade head elements, since that was the only available head element type for that implant.

### Primary endpoint

The primary endpoint was fracture union at 24 weeks postoperatively. In the FAS, there were a total of 93 patients in the TFNA group, of which 11 (11.8%) did not have an assessment for the measurement of fracture union at 24 weeks after surgery; there were a total of 92 patients in the PFNA-II group, of which 7 (7.6%) did not have an assessment for the measurement of fracture union at 24 weeks after surgery.

Based on analysis of the actual data collected from patients (non-missing data), all 82 patients in the TFNA group had fracture union, and all 85 patients in the PFNA-II group had fracture union, resulting in a fracture union rate of 100% at 24 weeks after surgery in both groups. When the Newcombe–Wilson scoring method was used, the fracture union rate difference between the TFNA group and the PFNA-II group was 0. The two-sided 95% CI [− 4.48%; 4.32%] was within the 10% threshold of non-inferiority, indicating that the TFNA group was not inferior to the PFNA-II group.

**Fig. 2:**
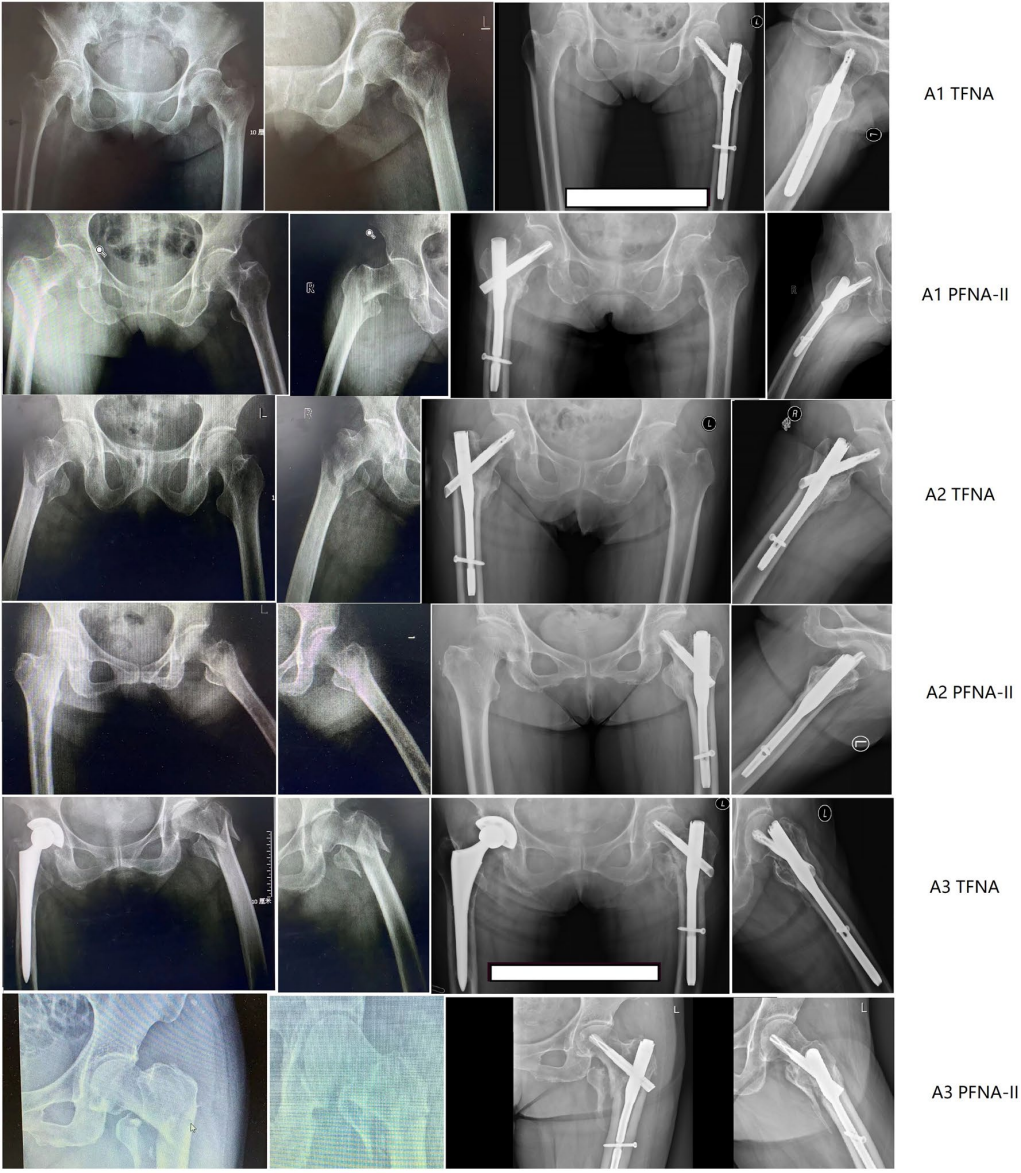
Examples of X-ray images pre-op and 24 weeks post-op for 3 OA classifications (31-A1, 31-A2 and 31-A3) in 2 groups. Abbreviations: *PFNA-II* Proximal Femoral Nailing Antirotation, *TFNA* Trochanteric Fixation Nail Advanced

Examples of each of the fracture classes and implantations of the TFNA and PFNA-II devices with comparable union are shown in Fig. 2.

### Secondary endpoints

Results from the Harris Hip Score, SF-12 (physical and mental components), and EQ-5D Health Index score are summarized in Table 4. There was a slight variation in sample size for each assessment due to missing data for some outcomes. The *p*-values represent a comparison of study vs. control groups, with some comparisons being the change from baseline, where indicated.

Harris Hip Score results for the 83 patients in the TFNA group with data showed a mean (SD) score at 12 weeks after surgery of 77.71±10.94 and a mean score for the 85 patients in the PFNA-II group at 12 weeks after surgery of 80.62±11.06. A Group t test was adopted for comparison of the TFNA and PFNA-II groups; the *p* value was 0.0882, indicating no statistically significant difference. The mean Harris Hip Score for the 82 patients in the TFNA group with data at 24 weeks after surgery was 86.03±10.42, and for the 85 patients in the PFNA-II group was 86.47±11.24. The *p* value was 0.7938, indicating no statistically significant difference. The score for both groups improved between 12 and 24 weeks (Table 4).

**Table 4:** Secondary endpoints: Harris Hip Score, SF-12 Physical and Mental, and EQ-5D Questionnaire results 12 and 24 weeks after surgery

Outcome scale	Baseline		12 Weeks			24 weeks		
	Study *N*=93	Control *N*=9	Study *N*^a^≥83	Control *N*^a^≥84	*p* value^b^	Study *N*^a^≥81	Control *N*^a^≥84	*p* value^b^
	Mean (SD)	Mean (SD)	Mean (SD)	Mean (SD)		Mean (SD)	Mean (SD)	
Harris Hip								
0–100			77.71 (10.94)	80.62 (11.06)	0.0882	86.03 (10.42)	86.47 (11.24)	0.7938
SF-12 Physical								
0–100	34.85 (7.53)	35.24 (8.26)	37.59 (6.62)	38.69 (6.71)	*0.2948*	41.41 (7.66)	41.20 (6.93)	*0.8110*
SF-12 Mental								
0–100	54.07 (12.66)	54.27 (12.80)	54.93 (9.82)	55.10 (9.64)	*0.6672*	56.00 (9.26)	55.09 (9.90)	*0.6032*
EQ 5D HI								
0–1			0.63 (0.16)	0.66 (0.13)	0.1817	0.73 (0.17)	0.72 (0.15)	0.5865

^Abbreviations: *EQ-5D HI* EuroQuol 5D Health Index, *n* number, *SD* standard deviation, *SF* short form^

^a^There was slight variation in sample size because of missing data for some patients on respective outcomes

^b^Comparison of Study vs. Control groups; p values in italics indicate the comparison of change from baseline outcomes

SF-12 total physical score results for the 84 patients in the TFNA group with data showed a mean (SD) total physical score at 12 weeks after surgery of 37.59±6.62; the mean total physical health score of the 84 patients in the PFNA-II group was 38.69±6.71. The *p* value was 0.2948, indicating no statistical significance between the two groups. At 12 weeks after surgery, the total mental health score of the 84 patients in the TFNA group with data was 54.93±9.82; the total mental health score of the 84 patients in the PFNA-II group was 55.10±9.64. The *p* value was 0.6672, indicating no statistical significance between the two groups.

At 24 weeks after surgery, the mean total physical health score of the 81 patients in the TFNA group with data was 41.41±7.66; the mean total physical health score of the 84 patients in the PFNA-II group was 41.20±6.93. The *p* value was 0.8110, indicating no statistical significance between the two groups. At 24 weeks after surgery, the total mental health score of the 81 patients in the TFNA group with data was 56.00±9.26; the total mental health score of the 84 patients in the PFNA-II group was 55.09±9.90. The *p* value was 0.6032, indicating no statistical significance between the two groups.

EQ-5D Health Index scores are also summarized in Table 4. At 12 weeks after surgery, the mean (SD) change in health index of 84 patients in the TFNA group with data was 0.63±0.16; the change of health index of 84 patients in the PFNA-II group was 0.66±0.13. The *p* value was 0.1817, indicating no statistical significance between the two groups. At 24 weeks after surgery, the mean (SD) change in health index of 81 patients in the TFNA group with data was 0.73±0.17; the change in health index of 84 patient in the PFNA-II group was 0.72±0.15. The *p* value was 0.5865 indicating no statistical significance between the two groups.

### Safety

There were no revisions (component removal for any reason) or re-operations (secondary reoperation of the fractures site for any reason) during the post-operative period at 24 weeks in either study cohort. Adverse events were collected regardless of relationship to the device or procedure. A total of 22 serious adverse events occurred in 18 patients (19.4%) in the TFNA group, and a total of 21 serious adverse events occurred in 16 patients (17.4%) in the PFNA-II group. When classified according to relationship, there were no SAEs related to the device in either group. The SAE rate difference between the two groups was 1.96% (*p*=0.7302) (Table 5). In the TFNA group, there was one SAE assessed as unlikely to be related to the procedure and one SAE assessed as possibly related to the procedure. In the PFNA-II group, there was one SAE assessed as unlikely to be related to the procedure (Table 6). Regarding severity of SAEs, among the 18 patients in the TFNA group, five were mild, (27.8%), eight were moderate (44.4%), and five were severe (27.8%). Among the 16 patients in the PFNA-II group, 3 were mild (18.8%), 7 were moderate (43.8%), and 6 were severe (37.5%) (Table 6).

**Table 5:** Summary of SAEs

System organ class preferred term	Study Group * N* = 93		Control Group *N* = 92	
	No of events	No. of patients (%^a^)	No. of events	No. of patients (%^a^)
Nervous system diseases	5	4 (4.3)	3	3 (3.3)
Hemiplegia	1	1 (1.1)	0	0
Cerebral infarction	1	1 (1.1)	1	1 (1.1)
Syncope	1	1 (1.1)	0	0
Cerebral thrombosis	2	1 (1.1)	0	0
Cerebral hemorrhage	0	0	1	1 (1.1)
Vascular dementia	0	0	1	1 (1.1)
Surgeries/medical operations	3	3 (3.2)	3	3 (3.3)
Rehabilitation treatment	3	3 (3.2)	2	2 (2.2)
Bone surgery	0	0	1	1 (1.1)
Injuries/poisoning/complications	2	2 (2.2)	3	3 (3.3)
Post-procedural complication	1	1 (1.1)	0	0
Femoral fracture	1	1 (1.1)	1	1 (1.1)
Postoperative hematoma	0	0	1	1 (1.1)
Pelvic fracture	0	0	1	1 (1.1)
Respiratory/thoracic/mediastinal	2	2 (2.2)	5	3 (3.3)
Pulmonary inflammation	2	2 (2.2)	3	2 (2.2)
Abnormality of respiration	0	0	1	1 (1.1)
Respiratory failure	0	0	1	1 (1.1)
Cardiac disorders	3	2 (2.2)	2	2 (2.2)
Coronary stenosis	1	1 (1.1)	0	0
Cardiac asthma	1	1 (1.1)	0	0
Atrial fibrillation	1	1 (1.1)	1	1 (1.1)
Unstable angina pectoralis	0	0	1	1 (1.1)
Infections/infectious diseases	2	2 (2.2)	0	0
Upper respiratory infection	1	1 (1.1)	0	0
Bronchitis	1	1 (1.1)	0	0
Gastrointestinal disorders	2	2 (2.2)	0	0
UGI hemorrhage	2	2 (2.2)	0	0
Metabolism/nutrition disorders	1	1 (1.1)	1	1 (1.1)
Poor diabetes control	1	1 (1.1)	0	0
Hypokalemia	0	0	1	1 (1.1)
Systemic disease	1	1 (1.1)	1	1 (1.1)
Fever	1	1 (1.1)	1	1 (1.1)
Renal/urinary system diseases	1	1 (1.1)	0	0
Chronic kidney disease	1	1 (1.1)	0	0
Skin/subcutaneous disorders	0	0	1	1 (1.1)
Psoriasis	0	0	1	1 (1.1)
Hepatobiliary system diseases	0	0	2	2 (2.2)
Chronic cholecystitis	0	0	1	1 (1.1)
Abnormal liver function	0	0	1	1 (1.1)

Serious adverse event	Study group	Control group	Statistics	*p* value
Total number of SAEs				
Number of patients	93	92	0.1189	0.7302^b^
Yes	18 (19.4%)	16 (17.4%)		
No	75 (80.6%)	76 (82.6%)		

SAE rate difference^c^ (study group-control group) and 95% CI: 1.96% [− 10.27%,14.20%]

Abbreviations: *CI* confidence interval, *N* number, *SAE* serious adverse event, *UGI* upper gastrointestinal

^a^The total number of SAEs refers to the number of patients suffering from SAEs with the SAEs occurring in the patient at least one time, which is considered as “Yes”

^b^Likelihood ratio Chi-square test

^c^Difference in SAE rate and 95% confidence interval were analyzed by continuously corrected Wald test

**Table 6:** Analysis of severity of SAEs and conditions related to investigational devices (calculated according to number of patients)

Index	Study group (*N* = 93)	Control group (*N* = 92)	*p* value
Severity of SAE^a^			0.9056^b^
Mild	5 (27.8%)	3 (18.8%)	
Moderate	8 (44.4%)	7 (43.8%)	
Severe	5 (27.8%)	6 (37.5%)	
Correlation between SAEs and device			NA
Unrelated	18 (100%)	16 (100%)	
Unlikely	0	0	
Possibly related	0	0	
Probably related	0	0	
Definitely related	0	0	
Correlation: SAE and surgery			1.000^b^
Unrelated	16 (88.9%)	15 (93.8%)	
Unlikely	1 (5.6%)	1 (6.3%)	
Possibly related	1 (5.6%)	0	
Probably related	0	0	
Definitely related	0	0	

Abbreviations: *N* number, *SAE* serious adverse event

^a^The highest severity or correlation was used for analysis if multiple SAEs occurred in a single patient

^b^Fisher’s exact probability test

One patient had a TFNA helical blade cut-out 63 days after implant due to a fall, reported as an SAE with causation related to the fall and not the device. The investigator had suggested a revision, but the patient refused, and surgery was not performed. There were no revisions or reoperations in either group.

## Discussion

In terms of the primary outcome, it was found that the healing rate based on the collected patient data (non-missing data) was 100% in both the TFNA group and the PFNA-II group. We believe that our high degree of fracture union was at least partly attributable to the stringent exclusion criteria shown in Table 1, which was designed to reduce cofounding factors.

Regarding secondary outcomes, information was collected for the revision and reoperation rates at 24 weeks after surgery, as well as the SF-12, Harris Hip, and EQ-5D Scores. There were not revisions or reoperations in either group. Clinical outcome scores were statistically indistinguishable, indicating no signification differences between either implant. In addition to these clinical outcomes, the apparent equivalence of surgical markers such as blood loss and surgery time supported the non-inferiority of the TFNA compared to the PFNA-II.

During the study, all intra- and post-operative AEs were recorded and evaluated for relationship to the device or procedure. With a total of 18 and 16 SAEs occurring in the TFNA and PFNA-II groups, respectively, it was shown that the safety profile of TFNA demonstrated no significant difference from the PFNA-II. No device-related adverse events were identified in the clinical study.

Regarding the operability of both the TFNA and PFNA-II devices, surgeons’ assessments of the two products across various stages of handling were found to be virtually identical. Because differences in healing rates and Harris scores and safety between blades and screws were found to be insignificant, the selection of blades or screws depended upon the preference of surgeons. The static locking option was not collected because the surgeons only used that option when the AO classification of the fracture was A3 or if there were other unstable fractures, less than 10% of the patients studied (Table 2). While not statistically significant, possible improvement was observed in the TFNA group with regard to the parameters including “determine nail application position,” “inserting the nail,” “proximal locking,” “distal locking,” and “placing end cap.” This evaluation provided substantial support for the ability of surgeons familiar with the PFNA-II system to adapt to the TFNA device. While not statistically significant, it also suggested a potential enhancement of the surgical experience, presumably due to the new design features introduced by the TFNA implant and instrumentation. With results from both the PPS and FAS analyses demonstrating non-inferiority, the FAS results were considered to be a sufficient representation of the apparent equivalence of the two nailing systems.

The average age of our patients was 77.8 years for the TFNA group and 74.1 years for the PFNA-II group, in excellent agreement with the average age of 77.1 of the 190,560 patients confirmed as having various classes of hip fracture obtained from the Urban Employee Basic Medical Insurance and Urban Resident Basic Medical Insurance databases, covering over 95% of the whole urban population of China [11]. Our follow-up period of 6 months was in agreement with the time span reported in other studies of the PFNA system (1,5), limiting number of drop-outs that could have occurred over a longer follow-up.

In agreement with our findings, Yoon et al. reported an excellent outcome for the TFNA concerning safety and the surgical treatment of proximal femur fractures in Korea. Specifically, 100% bone union was observed with no early postoperative complications, no implant failures, and no cases of revision surgery [12]. Matsumura et al. found that internal fixation using the TFNA and PFNA-II devices of similar size for Japanese patients with trochanteric hip fractures (even those with unstable conditions) resulted in a union rate of 98% with minimal blood loss and no cases of nail jamming [13]. Unsay et al. reported that early experience in India with the TFNA demonstrated that all fractures healed uneventfully with no distal anterior cortical impingement or perforation; this descriptive study suggested that this cephalomedullary nail was at least comparable to preceding proximal femur nail devices in terms of fixation [14].

Compared to outcome measures of the PFNA-II, PFNA, InterTAN, and Gamma intramedullary nails, the performance of the TFNA in several parameters appears to be equivalent to several values reported in the literature. Table 7 demonstrates a close matching of Harris Hip Scores, the physical and mental components of the SF-12 questionnaire, and revision surgeries and reoperations across all products. In these aspects, our experimental data have shown that the TFNA device is expected not to show significant differences from other cephalomedullary nails in the market place.

**Table 7:** Performance comparisons of TFNA, PFNA, PFNA-II, InterTan, and Gamma Intramedullary nails in the treatment of intertrochanteric fractures of the proximal femur

Study	HHS	SF-12P	SF-12 M	Revision rate	Reoperation rate
Current					
TFNA	**86.03**	**41.41**	**56.00**	**0/93**	**0/93**
PFNA-II	**86.47**	**41.20**	**55.09**	**0/92**	**0/93**
Singh (29)					
PFNA-II	79.73	43.56			2/30
Yu (30)					
PFNA-II	83.8				
InterTAN	82.6				
Saudan (31)					
PFN					6/79
Adams (32)					
Gamma				12/203	
Loo (1)					
PFNA				0/21	
PFNA-II				0/41	
Imerci (33)					
PFNA	78.06			1/16	
Schipper (34)					
PFN	66.8				
Gamma	69.5				
Zhang (35)					
PFNA-II	82.6				3/46
InterTan	80.2				2/47
Oku (36)					
PFNA	74				2/33
Seyhan (37)					
PFNA	80.93				0/43
InterTan	82.43				0/32
Zehir (38)					
PFNA	75.87				9/96
InterTan	71.26				5/102
Zhang (39)					
PFNA	72.4				16/115
InterTan	72.2				4/124
Zhang (40)					
PFNA	78.01				6/88
InterTan	79.97				0/86

Bold numbers are the outcomes from the current study

Abbreviations: *TFNA* Trochanteric Fixation Nail Advanced, *PFNA-II* Proximal Femoral Nailing Antirotation, *PFN* Proximal Femoral Nail, *HHS* Harris Hip Score, *SF-12P* SF-12 Physical Component Score, *SF-12M*  SF-12 Mental Component Score

The absence of implant breakages in our findings may be related to the paucity of type A3 fractures included in our study groups. In contrast with our prospective study, Lambers et al. [15] reported 16 cases of TFNA nail breakage over a timeline of two years in their retrospective study. In the Lambers study, the initial fracture pattern in 75% of the cases was found to be reverse oblique fractures—precisely the kind that are challenging to reduce, such that the healing process can be delayed. This possibility was supported by the finding in a retrospective study of 176 Asian patients in which individuals with a type A3 fracture were 2.9 times more likely to experience a fracture fixation complication than patients with an A2 fracture. In just a third of the incidents attributable to the fracture class, the complication relation to the implant device itself was deemed to be possible rather than probable or definite [16]. Finally, in Lambers et al., the total number of TFNAs implanted in the three trauma hospitals utilized was not indicated; therefore, an incidence rate could not be determined. No breakage in cases of simple pertrochanteric fracture patterns was seen [15].

Further support regarding the safety of the TFNA device was obtained through a study of retrieved data from a large US healthcare database containing inpatient discharges from geographically diverse hospitals in both urban and rural areas. Since 2012, the database has received contributions of data from over 700 hospitals. The study compared data from the TFNA to a comparison group, which included the Stryker Gamma3 and Zimmer Natural Nail (non-TFNA group). During the study period, implantations with 14,370 TFNA nails were identified and compared with implantations of 8260 non-TFNA nails. When the risk data were balanced, the risk of breakage in 18 months was equivalent in subgroup analyses involving pertrochanteric factures or subtrochanteric fractures [17].

This was the first randomized controlled trial demonstrating the noninferiority of fracture union rates for the TFNA system to an established device in China. The noninferiority approach to benchmarking offers an immediate comparison of implanted devices using a reference standard, featuring advantages compared to standard Kaplan–Meier analyses [18]. The possibility that there was a bias toward the null was unlikely due to the minimal numbers of missing data. The inclusion of both the FAS (intention-to-treat) and PPS (per protocol) analyses (latter data not shown) and the fact that their results were virtually identical avoids pitfalls associated with each method alone [19] and provides additional rigor to our conclusion that the TFNA device was not inferior [19, 20]. Furthermore, our comparison of outcomes of the TFNA against what could be deemed as a standard of care with the PFNA-II product is yet another measure to reduce the risk of bias [6]. Reporting of both the absolute and relative effect measures could be considered to be yet another strength of the study, based on the criteria for equivalence reported elsewhere [21]. Finally, the importance of demonstrating both statistical and clinical equivalence to validate a noninferiority has been described in the literature [22] and is included in our data.

## Limitations

The drawing from 9 major trauma centers involving multiple surgeons in a broad range of clinical settings suggests a greater external validity (generalizability) than more confined clinical trials, yet our investigation was designed to address regional regulatory requirements and therefore was restricted to an Asian population in a single country. In our study, 96% of the TFNAs and 98% of the PFNA II products were short nails, respectively. Although studies have compared the outcomes of short versus long nails applied to intertrochanteric femur fractures, results have shown no statistically significant difference in facture union^23^ as well as insignificant differences in reoperations or complication rates [23–25]. Our study group was relatively small, and the follow-up time of 24 weeks was limited, although the nearly identical outcomes in both experimental groups bode well for the study’s validity. Furthermore, the types of lesions studied were restricted with the exclusion of osteoporotic and combination bone fractures (indications for the TFNA), although our extended list of exclusions allowed our study group to be more homogenous with more clearcut classifications of bone fractures—a welcoming feature for more rigorous randomized clinical trials.

## Conclusions

In a randomized, blinded, and controlled noninferiority clinical trial, we have found that there was no significant difference in the safety and performance outcomes between the TFNA and PFNA-II devices investigated in this study. It can therefore be concluded that the TFNA is as efficacious and safe as the PFNA-II product to treat fractures of the proximal femur and femoral shaft.

## Data Availability

The data that support the findings of this study are available from Johnson & Johnson Medical (Shanghai) Ltd., but restrictions apply to the availability of these data, which were used under license for the current study, and so are not publicly available. Data are however available from the authors upon reasonable request and with permission of Johnson & Johnson Medical (Shanghai) Ltd.

## Abbreviations

AE: Adverse event
AO: Arbeitsgemeinschaft fur Osteosynthesefragen
CI: Confidence interval
EQ-5D: EuroQuol Quality of Life Index
FAS: Full analysis set
HI: Health Index
Hr: Hour
mL: Milliliter
OTA: Orthopedic Trauma Association
PFNA-II: Proximal Femoral Nailing Antirotation
PPS: Per Protocol Set
PT: Preferred term
SAE: Serious adverse event
SD: Standard deviation
SF: Short form
SOC: System organ class
TFNA: Trochanteric Fixation Nail Advanced
Ti-6 l-7Nb: Titanium-Aluminum-Niobium
TiMo: Titanium-Molybdenum
